# On the Administration of Intoxicating Doses of Alcohol in Traumatic Tetanus

**Published:** 1845-05

**Authors:** J. W. Stapleton

**Affiliations:** Trowbridge


*On the Administration of Intoxicating Doses of Alcohol in Traumatic Tetanus.* By J. W. Stapleton, Trowbridge.—Charles P——, a farmer's labourer, aged seventeen, sustained on the 24th Dec. last, a lacerated wound of the palmar surface, and outer edge of the carpus of the right hand. It was inflicted by a chaff-cutting machine, the blade of which had stripped off a flap of the integument, leaving an irregular contused edge; no tendinous, muscular, or nervous structures were involved, nor was the palmar fascia exposed. Those portions of the flap whose texture was destroyed were at once removed by the knife, and the rest was replaced and supported with adhesive straps, and a light bandage covered with the cold-water dressing. On the sixth day, the parts had nearly united, and a healthy granulating surface presented where there had been loss of substance. On the afternoon of the twelfth day, (having been dressed in the morning,) he came back to my surgery, complaining of intense pain, extending from the lower extremity of the sternum, backwards, to the spine. On stripping him, I found the sternum and lower ribs powerfully retracted by *tonic* spasm of the diaphragm, and much tension of the muscles of the neck and jaw. The opisthotonus, which was permanent, was at times increased by violent clonic spasms of the same muscles; when these subsided, the arched form of the neck remained unaffected by their remission. The wound was bathed in an offensive sanious discharge, and had become extremely painful.

As a first and obvious duty, I removed from the wound every source of pressure and irritation, applying a simple poultice, and had the patient put to bed. Three drops of croton oil were placed upon the tongue, and a blister laid along the whole course of the spinal column; and as soon as the bowels had acted, a grain of the acetate of morphia was given, and repeated every hour.

In three hours I visited him again. All the symptoms were increased; there was a deep hollow at the scrobiculus cordis, and excruciating pain throughout the diaphragmatic region. The muscles of the neck were perfectly rigid, and those of the dorsal and lumbar regions participating in the tetanic spasm, all the joints of the trunk of the body seemed anchylosed, so that when removed from the bed, he lay in the arms of those who carried him like a marble statue. He still referred all his pain to the diaphragm, and to the course of the recti. The abdomen was forcibly thrust forward, and was immovable during the respiratory act, which was maintained wholly by the thoracic muscles. No complaint was made of the hand from this time. Morphia was again given and continued throughout the night, without any mitigation of the symptoms.

On the following morning, the disease was evidently proceeding with rapidity to a fatal issue. The pulse was eighty-five, labouring and constricted; the heart's action tumultuous and irregular; the skin of the natural temperature, and the intellect unimpaired. I gave the following injection.—Oil of turpentine, one oz.; tincture of opium, two drachms; assafœtida mixture, twelve oz.; hop water, eight oz.; mix them for an injection. At the same time, two grains of morphia were sprinkled upon the surface of the blister, and the following medicines directed to be given every alternate hour—Take assafœtida, eight grains; Camphor, six grains; let them be made into a bolus to be taken immediately, and repeated every two hours; and with these take acetate of morphia, one grain; extract of belladonna, one grain; extract of conium, four grains; made into a pill to be taken one hour after the bolus, and repeated every two hours.

Four hours after this he was evidently worse. Blisters were now placed on the abdomen, over the recti muscles; and as the injection was retained, I gave him three drops of croton, which soon acted copiously, yet without any remission of the symptoms.

A fair trial of the opiates and antispasmodics had now been given; twenty-six hours having been fruitlessly occupied in their administration. The progress of the case had been uninterrupted from bad to worse. The patient lay immovably fixed, his weight resting on the back of the head and the sacrum; the trunk of his body between these two points forming an arch, whose concavity was towards the mattrass, and within which a child might have crept without touching him. He could not open his mouth further than to allow a pill to be dropped within the teeth; deglutition was difficult, and the lips were distorted into a horrible sardonic grin; his eyes glared from their sockets, and every feature was indicative of the extremity of anxiety and suffering. He was perfectly sensible, and would reply to questions, but always as briefly as he could, and begging to be left alone. Pulse 90, sharp and jerking; skin hot and dry; cheeks flushed. I make this detail to enable me to contrast this picture with that which was presently assumed.

As those remedies which experience and authority had sanctioned seemed utterly nugatory, and in the assurance of the speedy death of my patient if I could oppose no check to the progress of his frightful disease, it suddenly occurred to me to make the trial of producing profound intoxication. The most striking symptom in the pathology of this condition is, undoubtedly, the influence it possesses over the muscles of voluntary motion, and the excito-motory system generally. I conjectured, further, that by administering alcohol freely, it might, by entering the blood, *and independently of its intoxicating effect*, establish another condition of the nervous centres, *incompatible with the continuance of the tetanic spasm.* I had everything to hope and nothing to fear from its effects, if cautiously watched, and considering the hopeless condition of my patient, I could discover no record of the use of the remedy *so exhibited*, but therapeutics and analogy confirmed my suggestion, and directed me where experience and authority ceased to guide. The effect of alcohol in calming the subsultus of typhus is unquestioned. I determined, therefore, to be limited in its exhibition only by the appearance of the condition I desired to produce, or by the occurrence of any injurious symptoms that could be fairly traced to its use.

Being provided with a mixture of alcohol and water, in equal parts, I gave him at once six ounces of such mixture, and four ounces more in a quarter of an hour; myself sitting by to note the result. In twenty-five minutes the patient (for the first time since the attack) lay on his side, in a profound and tranquil sleep, without any appearance of apoplectic stertor or other cerebral congestion. Every muscle was in a state of quiet relaxation, and the sense of pain had vanished as if by a charm. The tumultuous action of the heart had ceased; the pulse had sunk to 60, and was fuller in volume; the features had resumed their natural expression, and the whole surface of the body was drenched in a profuse perspiration.

For seventy-two hours he was kept under the influence of the remedy, complaining of no pain, and without any return of opisthotonus or clonic spasm, the bowels acting kindly and frequently, under the use of croton oil, and the opiates being abandoned.

On the 16th day the alcoholic influence was withdrawn, as a test of his real condition, and from fear of cerebral injury. With a return of consciousness, the tetanic symptoms returned, though in a much less degree, and as instantly and perfectly disappeared when the alcohol was again exhibited.

On the evening of the 17th day the back became again arched; the breathing hurried. He was at this time able to answer questions, and to move in his bed; the clonic spasms suddenly supervened, and he expired after their subsidence, without a struggle or a single evidence of pain, apparently from exhaustion.

On reviewing this case, I think I am justified in assuming that, in failure of the usual sedative treatment, much excruciating suffering was prevented by the use of alcohol; and that it might have prevented the accession of the disease, or, at least, *its establishment in the constitution*, had it been adopted at an earlier period. The unfortunate termination of the case I am inclined to attribute rather to fatal collapse than to the tetanic paroxysm. My only regret is, that the early and most valuable period was lost to treatment.

It appears that in Mr. Potter's case the arched form was retained, and gradually increased *during* the exhibition of the hemp extract. In my own case no opisthotonus remained during the free administration of the alcohol.

In Dr. Hutchinson's case recovery was effected under the use of the belladonna. It had no effect in the case I have detailed. I commenced with grain doses of the extract combined with other sedatives, but the symptoms became more grave in their character with each dose, and before I had time to produce constitutional toleration of the remedy, it seemed hurrying to a fatal termination.

In this part of the country this disease is of very infrequent occurrence, but at the time of this case there were two others in this town, both of them idiopathic; one under my own care, the result of exposure to cold after a febrile attack, in a young woman of twenty-two. It yielded readily to blisters on the neck, and free purgation with the croton. Of the other case, which occurred in the practice of another gentleman, I have only learnt that recovery took place. I regard this concurrence of cases as remarkable, and am disposed to attribute it to some morbific state of the present humid atmosphere; the more so, regarding its frequent occurrence in fenny and miasmatic districts.

These details exceed the limit of my wishes and of your forbearance. Their only pretensions to publicity are their truth and their presumed utility.—*London Lancet.*

